# Gastrointestinal Microbiota & Symptoms of Depression and Anxiety in Anorexia Nervosa—A Re-Analysis of the MICROBIAN Longitudinal Study

**DOI:** 10.3390/nu16060891

**Published:** 2024-03-19

**Authors:** Jasmin Ketel, Miquel Bosch-Bruguera, Greta Auchter, Ulrich Cuntz, Stephan Zipfel, Paul Enck, Isabelle Mack

**Affiliations:** 1 Department of Psychosomatic Medicine and Psychotherapy, University Hospital Tübingen, 72076 Tübingen, Germany; 2 Centre of Excellence for Eating Disorders (KOMET), 72076 Tübingen, Germany; 3 Klinik Roseneck, Center for Behavioral Medicine, 83209 Prien am Chiemsee, Germany; 4 Forschungsprogramm für Psychotherapieevaluation im Komplexen Therapiesetting, Paracelsus Medical University (PMU), 5020 Salzburg, Austria

**Keywords:** microbiota, gastrointestinal, gut, anorexia, depression, anxiety, eating disorder

## Abstract

The microbiota–gut–brain axis may play a role in the pathophysiology of anorexia nervosa (AN). Here, the relationship between the gastrointestinal microbiota and symptoms of depression, anxiety, and eating disorder pathology in patients with AN before (*n* = 55) and after weight restoration (*n* = 44) was investigated by reanalyzing the data of the MICROBIAN study. The gastrointestinal microbiota was analyzed using 16S rRNA amplicon sequencing. Symptoms of anxiety disorder, depression, and the severity of the eating disorder were measured by validated questionnaires. All analyses were adjusted for the body mass index (BMI). Several significant findings between psychological parameters and the gastrointestinal microbiota were not evident after controlling for the BMI. No differences in alpha and beta diversity between groups of higher and lower symptom severity levels for depression and anxiety were found. Positive associations between species of *Blautia* and *Ruminococcus* and depression symptoms, and between the phylum Firmicutes and anxiety symptoms were observed after rehabilitation, respectively. A positive correlation was found between propionate and acetate levels and the reduction of depression severity during inpatient treatment. Accounting for the weight status when analyzing the relationship between psychological parameters and the gastrointestinal microbiota in patients with underweight is important since the BMI may be the driver for many observed changes.

## 1. Introduction

Anorexia nervosa (AN) is a mental disorder characterized by a self-induced loss of body weight and disturbed body image [1]. Because of the extreme fear of weight gain, AN is accompanied by behaviors to maintain or further reduce the low body weight, such as restrictive eating, increase in energy expenditure, and purging behaviors [1]. Difficulties in accepting treatment [2] and gastrointestinal complaints are common problems in the therapy of patients with AN [3]. To improve the high relapse rates of 50% in the long term, alternative therapy approaches besides psychotherapy are necessary [4].

The etiology of AN is still unclear. Environmental, psychosocial, and interpersonal factors can contribute to its development [5]. In addition, physiological mechanisms could be involved in the onset and progression of the disease, the gut microbiota could be one such factor [6].

Gut microbiota alterations are also connected with gastrointestinal disorders, such as inflammatory bowel disease and colon cancer [7]. In addition, associations of alterations in the gastrointestinal microbiota with atopic disease [8], diabetes [9], and metabolic syndrome [10] are underlining its systemic influence on human health. In line, differences in the gastrointestinal microbiota composition in AN versus healthy, normal-weight participants have been reported [11,12,13,14]. However, the gut microbiota composition in AN appears not to negatively affect weight gain during refeeding. Glenny et al. [15] showed, that stool transplantation of patients with AN into germ-free mice resulted in similar weight gain when compared to that of animals which received stool transplantation of healthy normal-weight donors. This finding was recently repeated [16]. However, the latter study found that during weight restriction, animals receiving stool transplants from healthy donors performed better than those receiving fecal transplants from patients with AN.

Interestingly, the gastrointestinal microbiota could affect psychological processes via the gut–brain axis in AN. The gut–brain axis refers to bidirectional communication between the enteric and central nervous systems. It has an influence on both brain and gastrointestinal functions in multiple ways. The gut microbiota sends neuronal, immunological, and endocrine signals to the central nervous system [17]. In turn, the central nervous system has an influence on intestinal microbes through virulence gene expression and modulation of gut functions, such as motility and secretion [17]. Furthermore, the enteric nervous system affects the gut microbiota via alterations of immunological mechanisms, permeability as well as mucus secretion, and gut motility [17]. Therefore, the reported alterations of the gastrointestinal microbiota of patients with AN could influence the onset and progression of changes in brain function and behavior in this disease.

Short-chain fatty acids (SCFAs), produced by bacterial fermentation of dietary fiber, are linked to neurological and psychiatric disorders [18]. Bacterial dysbiosis alters SCFA concentrations, affecting various aspects of host health, including neurotransmitter production, mitochondrial function, immune system, lipid metabolism, and gene expression. This consequently influences the central nervous system [18]. Associations between gastrointestinal microbiota, SCFAs, and neuropsychiatric diseases, such as depression and anxiety, are debated [18,19,20]. Metagenomic analysis in AN suggests a role for the gastrointestinal microbiome in its pathogenesis, with increased functional modules for neurotransmitter degradation observed in patients compared to healthy controls, potentially impacting mood and appetite [16].

Due to the high prevalence of comorbidities of depression and anxiety disorders in AN [5] and the link between microbiota and psyche, several small studies have already examined this connection in patients with AN resulting in an inconsistent outcome. Kleiman et al. [21] discovered a negative association between levels of depression and microbial richness, whereas Schulz et al. [22] reported no correlation between bacterial richness and diversity with symptoms of depression and anxiety. Borgo et al. [23] also observed a negative relation between depression scores and *Clostridium* spp. as well as a negative association between fecal butyrate concentrations and depression and anxiety disorder. Likewise, also Castellini et al. [24] discovered a relationship of the bacterial community with butyric acid and anxiety in patients with AN. At the same time, both authors pointed out the significant correlation of depression [23] and anxiety [23,24] with the body mass index (BMI) in these studies. For the general population, a U-shaped curve for the relation between BMI and depression has been reported [25,26]. Regarding the underweight range, this implies that depression symptoms are high in severe underweight and decline with increasing body weight. Beyond that, multiple studies reported a relationship between the gastrointestinal microbiota and the severity of the eating disorder pathology [16,21,23,24].

In summary, there is anecdotal evidence for a link between the gastrointestinal microbiota as a modulator of the gut-brain axis affecting anxiety and depression in patients with AN. However, it is unclear whether these effects are due to the pathological influence of the microbiota or to the severity of the eating disorder symptoms and malnutrition. Therefore, we aimed to investigate the relationship between the gastrointestinal microbiota and symptoms of depression, anxiety, and eating disorder pathology in patients with AN before and after weight restoration by reanalyzing the data of the MICROBIAN study [27]. We hypothesized that measurable differences in microbial richness, diversity, and community structure between patients with AN with higher and lower levels of symptom severity in depression, anxiety, and eating disorders are influenced by the state of malnutrition and starvation, namely the BMI.

## 2. Materials and Methods

The MICROBIAN study investigated the relationship between the gastrointestinal microbiota and SCFAs gastrointestinal complaints and diet in patients with AN in comparison with healthy controls cross-sectionally and, longitudinally before and after weight restoration. At the time of analysis in 2015 and publication in 2016 [27], the questionnaire data on depression, anxiety, and eating disorder psychopathology for patients with AN were not available. In this re-analysis, we extend the findings from the original results by investigating the relationship between the gastrointestinal microbiota, depression, anxiety, and eating disorder psychopathology in patients with AN in the longitudinal approach. For healthy controls, no psychological data are available, which is the reason why they are not included in this re-analysis.

### 2.1. Ethics and Registrations of the Study

The experiments were conducted in accordance with the relevant guidelines and regulations. The Ethics Committee of the University Hospital Tübingen, Germany approved the study protocol (No. 429/2011BO2) and is registered at the German Clinical Trials Register (DRKS00005124). Participants were informed about the study’s purpose during the admission interview and were asked to provide written consent prior to inclusion. In the case of minors (<18 years of age), the parents were contacted by telephone and had to provide written informed consent.

### 2.2. Design and Population

The study population comprised women with AN, participating in an inpatient treatment program according to eating disorders guidelines [28] at the Schön Klinik Roseneck, Prien, Germany. The main aim of this program was to improve eating habits and to gain body weight. AN was diagnosed according to the Diagnostic and Statistical Manual of Mental Disorders, Fourth Edition [29] and the International Statistical Classification of Diseases and Related Health Problems 10 [30]. Patients with AN aged between 14 and 39 years with bulimic, purging, or atypical subtypes and BMI < 18 kg/m^2^ or <10% of the expected body weight in adolescents were included. Exclusion criteria were the intake of antibiotics within the previous eight weeks, having severe somatic diseases, or limited German language skills. Data and stool sampling in patients with AN was performed at the beginning (T1) and at the end (T2) of their inpatient stay.

### 2.3. Microbiota and SCFA Analysis

For the analysis of the microbiota and its metabolites, stool samples were collected, and stored, DNA isolated and amplicon library prepared, as well as SCFA analysis performed, as published in detail by Mack et al. [1].

Samples were analyzed by amplicon sequencing on an IlluminaMiSeq instrument (Illumina Inc., San Diego, CA, USA) by V4 16S rDNA according to this protocol [31]. A total of 22,133,491 reads corresponding to the V4 region were produced. The pre-processing of sequencing data, using an in-house pipeline based upon DADA2 [32] running on R version 4.1.0 [33] and rmarkdown [34,35,36], consisted of the following steps: reads filtering, identification of sequencing errors, dereplication, inference and removal of chimeric sequences [37]. The length of the raw reads was detected using HTSeqGenie [38] and sequence manipulation was done using Biostrings [39]. In order to assign taxonomy, DADA2 was used to annotate up to the species level using the database SILVA 138 version 2 [40]. Data were expressed as Amplicon Sequence Variants (ASVs). Decontam was used with either setting, which combines the two statistical methods prevalence and frequency for the identification of contamination in marker-gene and metagenomics data [41]. The tidyverse package was used for data manipulation and figure generation [42]. The phylogenetic tree was generated by midpoint rooting using the phangorn package [43]. There were 4360 ASVs in the pre-processed file, which was saved in the phyloseq format [44].

An online shiny app was used for ASV filtering [45]. All with a read count less than 839 (0.01%) and appearing in less than 8 samples (5%) were removed. A total of 376 ASVs were maintained for downstream analysis. There were 99 unique genera and 72 unique species in the final phyloseq. About 91.38% of ASV was removed.

For downstream analysis, R version 4.2.3 was used [46]. Alpha diversity indices (Shannon index, Chao1 index, inverse Simpson index, gini Simpson index) were estimated using the package microbiome [47]. Beta diversity distances were calculated by the packages phyloseq (unweighted and weighted UniFrac) [44] and vegan (Bray–Curtis index) [48]. Before calculating the alpha diversity indices and Bray–Curtis distance, data were aggregated on genus level for noise reduction. As the analysis of the Bray–Curtis and the weighted Unifrac distance showed similar results, only findings of the Bray–Curtis distance are reported below. Distance-based redundancy analysis (db-RDA) was performed with the capscale function in vegan [48]. Whereas for visualization and the Principal Coordinate Analysis (PCoA) the R package MicroViz version 0.10.10 [49] and GraphPad Prism version 9.4.1 for Windows [50] were used.

### 2.4. Psychological Parameters

For assessing psychological parameters, several self-report questionnaires were completed at the beginning and at the end of the inpatient stay. Therefore, the severity of depression symptoms was measured by the Patient-Health-Questionnaire-9 (PHQ-9) [51] and the severity of anxiety symptoms was quantified by the Generalized Anxiety Disorder Questionnaire (GAD-7) [52]. In addition, the pathology of eating disorders was assessed by Eating Disorder Inventory-II (EDI-II) [53]. As part of the clinical routine, the Beck depression inventory-II (BDI-II) [54], the Brief Symptom Inventory (BSI) [55], and the Structured Interview for Anorexia and Bulimia Nervosa (SIAB) [56] were also completed. These questionnaires showed a high correlation between each other (PHQ-9 and BDI-II: *r*(97) = 0.87, *p* < 0.001; PHQ-9 and BSI Scale 4: *r*(97) = 0.82, *p* < 0.001; BDI-II and BSI scale 4: *r*(97) = 0.8, *p* < 0.001; GAD-7 and BSI Scale 5: *r*(97) = 0.7, *p* < 0.001; EDI-II and SIAB: *r*(97) = 0.86, *p* < 0.001). For this reason, only findings for depression measured by PHQ-9, anxiety measured by GAD-7, and eating disorder measured by EDI-II are reported in the following. Results of analysis with BSI, BDI-II, and SIAB are provided in the Supplementary Material Tables S1–S13.

Manuals of questionnaires were used to classify depression and anxiety symptom severity (depression: 0–4 = minimal, 5–9 = mild, 10–14 = moderate, 15–21 = severe depression; anxiety: 0–4 = minimal, 5–9 = mild, 10–14 = moderate, 15–21 = severe anxiety) [51,52]. To detect clinically relevant improvements in symptom severity in depression and anxiety, minimal clinically important differences (MCIDs) were applied. Therefore, an improvement of four points in depression or anxiety symptom severity scores was set as MCID [57].

To enable the comparison between groups with higher and lower symptom severity of depression, anxiety, and eating disorders as well as with higher and lower improvement of these symptoms between T1 and T2, median splits were used. Using predefined thresholds or categories for creating such groups according to the manuals of the questionnaires was not possible since this led to extremely unequal, non-comparable group sizes. The values used for median splits are provided in Table 1 and Table 2.

### 2.5. Dietary Intake

Dietary intake was reported in detail by Mack et al. [27]. In short, intake was assessed at the beginning and at the end of the inpatient stay. During hospitalization, patients with AN ate specific diets designed for AN with 2000 to 3050 kcal/d. The energy intake was provided by 15% of proteins, 45% of fat, and 40% of carbohydrates.

### 2.6. Statistics

To estimate the consistency of the used questionnaires, their correlation was measured by Spearman rank’s correlation. Normal distribution was assessed with the Shapiro–Wilk test. Differences in BMI, age, depression, anxiety, and eating disorder symptoms between T1 and T2 were estimated by paired *t*-test for parametric and paired Wilcoxon-signed rank test for non-parametric data. For measuring the correlation of alpha diversity, absolute abundance of taxa on phylum, family, and genus levels, and SCFAs with the symptom severity of depression, anxiety, and eating disorder and their improvements between T1 and T2, Spearman rank’s correlation was used, too. In addition, linear models were used to adjust the relationships between taxa and symptom severities for BMI. *p*-values of correlation between taxa at family and genus level and symptom severity of psychological parameters were False Discovery Rate (FDR) adjusted [58]. Therefore, FDR < 0.15 was considered as statistically significant. Testing for group differences was conducted by the Mann–Whitney U test and Permutational analysis of variance (PERMANOVA). Permutational Multivariate Analysis of Covariance (PERMANCOVA) was used to adjust the analysis to the BMI [59].

## 3. Results

### 3.1. Characteristics

Fecal samples were analyzed from 55 female patients with AN at the beginning of the inpatient treatment (T1) and a second time from 44 female patients with AN after inpatient treatment (T2). The mean duration of hospitalization was 14.0 ± 6.8 (min–max: 4 –43) weeks. At T1, 39 patients could be assigned to the restrictive AN type (mean BMI: 14.94 ± 1.42) and 16 patients to the binge/purge type (mean BMI: 16.46 ± 1.3). Both groups differed significantly in BMI (*z* = 0.7, *p* = 0.002). As previously reported by Mack et al. [27], nutrient supply differed between normal-weight individuals and patients with AN, with lower energy and macronutrient intakes in patients with AN before inpatient treatment and higher fat, energy, and fiber intakes in patients with AN during weight gain.

The main characteristics are shown in Table 3. On average, patients with AN are characterized by a moderate level of anxiety and depression severity at T1. During inpatient treatment, 57% of patients with AN demonstrated clinically significant improvement in anxiety severity, and 80% of patients with AN improved clinically significant in depression severity according to MCIDs.

### 3.2. Alpha Diversity

**Figure 1:**
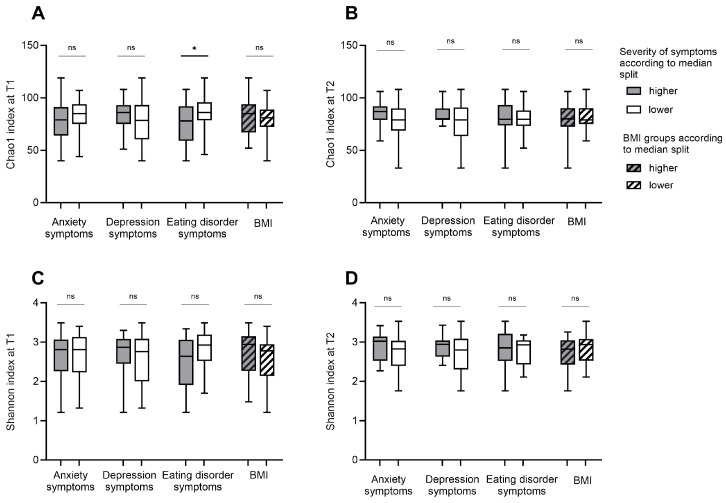
Alpha diversity in patients with Anorexia Nervosa in relation to body weight status and the severity of symptoms for eating disorder pathology, anxiety and depression before (T1) and after weight rehabilitation (T2): Microbial richness (Chao1 index; (**A**,**B**)) and diversity (Shannon index; (**C**,**D**)) are shown for groups with higher and lower severity of anxiety, depression and eating disorder pathology symptoms and for groups with higher and lower body mass index (BMI) at T1 and T2. Grouping was based on median splits of the symptom severity and BMI scores. Lower BMI groups were defined as BMI ≤ 15.3 at T1 and BMI ≤ 17.7 at T2. Higher BMI groups were defined as BMI > 15.3 at T1 and > 17.7 at T2. A *p* < 0.05 was considered as statistically significant. * indicates a *p* < 0.05, and ns indicates not significant.

**Figure 2:**
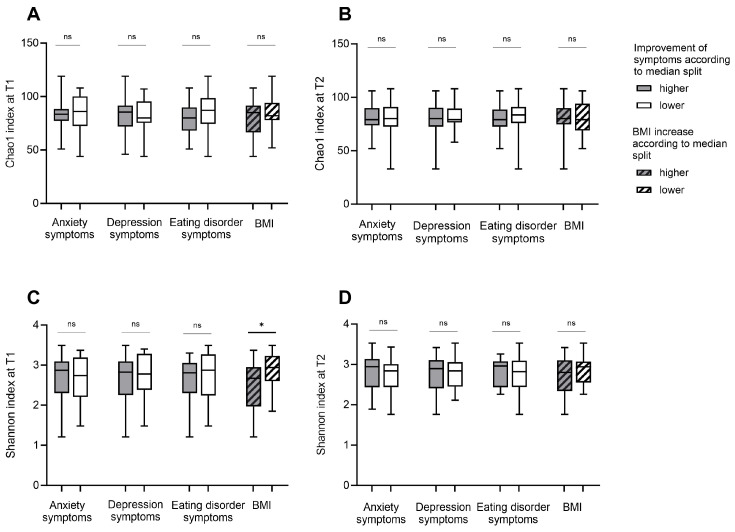
Alpha diversity in patients with Anorexia Nervosa in relation to the degree of improvement of body weight, symptoms for eating disorder pathology, anxiety and depression during weight rehabilitation: Microbial richness (Chao1 index; (**A**,**B**)) and diversity (Shannon index; (**C**,**D**)) are shown for groups with higher and lower improvement of anxiety, depression and eating disorder pathology symptoms and for groups with higher and lower increase in body mass index (BMI) during inpatient treatment. Grouping was based on median splits of the improvement in symptom severity scores and BMI scores between measurements before (T1) and after weight rehabilitation (T2). BMI groups with a higher increase during inpatient treatment were defined as BMI increase > 2 and groups with a lower increase during inpatient treatment were defined as BMI increase ≤ 2. A *p* < 0.05 was considered as statistically significant. * indicates a *p* < 0.05 and ns indicates not significant.

Microbial richness (Chao 1 index) and microbial diversity (Shannon index, inverse Simpson index, and gini Simpson index) were compared in groups with higher versus lower symptom severity according to the median split (Figure 1, Table S1). Since the outcomes for all three microbial diversity indices were identical, the Shannon index is reported as representative of the findings in Figure 1 and Figure 2. The inverse and gini Simpson indices data are shown in Tables S1–S3. Groups for anxiety and depression at T1 and T2 did not differ. A significant difference was observed at T1 in the microbial richness between groups with higher and lower eating disorder symptom severity. Considering the correlation of alpha diversity indices and psychological parameters, similar results were produced, indicating a negative correlation between the microbial diversity and eating disorder symptom severity at T1 (*r* (53) = −0.27, *p* = 0.047, Table S2).

Looking at the changes in psychological scores between T1 and T2, no differences in microbial diversity and richness between groups with higher and lower improvement of anxiety, depression, and eating disorder symptoms emerged (Figure 2, Table S3).

### 3.3. Beta Diversity

The dissimilarity in taxonomic composition (beta diversity) of patients with AN differed between T1 and T2 (Bray–Curtis: *z* = 1.16, *p* < 0.001; unweighted Unifrac: *z* = 1.52, *p* < 0.001). Indicating a decrease in beta diversity distances during inpatient treatment (Bray–Curtis: M(T1) = 0.56, M(T2) = 0.53; unweighted Unifrac: M(T1) = 0.48, M(T2) = 0.44).

**Figure 3:**
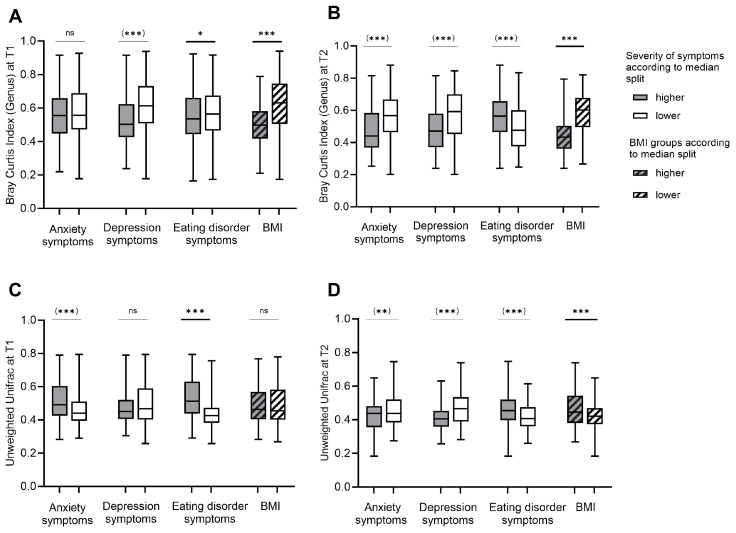
Beta diversity in patients with Anorexia Nervosa in relation to body weight status and the severity of symptoms for eating disorder pathology, anxiety and depression before (T1) and after weight rehabilitation (T2): Bray–Curtis (**A**,**B**) and Unweighted Unifrac (**C**,**D**) distances are shown for groups with higher and lower severity of anxiety, depression and eating disorder pathology symptoms and for groups with higher and lower body mass index (BMI). The matrices were calculated as pairwise distances between samples from different subjects in the same group. Grouping was based on median splits of the symptom severity scores and BMI scores. Lower BMI groups were defined as BMI ≤ 15.3 at T1 and BMI ≤ 17.7 at T2. Higher BMI groups were defined as BMI > 15.3 at T1 and BMI > 17.7 at T2. Statistical tests were performed using the Mann–Whitney U test and Permutational Multivariate Analysis of Covariance (PERMANCOVA) to adjust for BMI as a covariate. Results in parentheses indicate non-significant results after adjustment for BMI using PERMANCOVA. *p* < 0.05 was considered as statistically significant. * indicates *p* < 0.05, ** indicates *p* < 0.01, *** indicates *p* < 0.001 and ns indicates not significant.

As visualized in Figure 3, analysis of group differences using the Mann–Whitney U test revealed lower beta diversity indices in patients with lower severity of depression. In addition, differences in beta diversity between groups with higher and lower severity of anxiety and eating disorder symptoms were shown (Table S4). After adjusting the data for BMI using PERMANCOVA, no evidence for differences in beta diversity scores between groups with higher and lower severity of depression and anxiety symptoms was found (Tables S5–S7).

**Figure 4:**
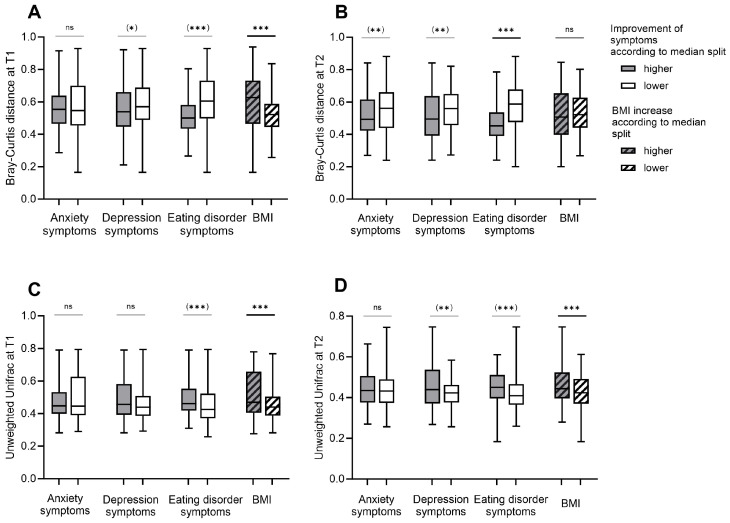
Beta diversity in patients with Anorexia Nervosa in relation to the degree of improvement of body weight, symptoms for eating disorder pathology, anxiety, and depression during weight rehabilitation: Bray–Curtis (**A**,**B**) and Unweighted Unifrac (**C**,**D**) distances are shown for groups with higher and lower improvement in anxiety, depression and eating disorder symptoms and for groups with higher and lower increase in body mass index (BMI) during inpatient treatment. The matrices were calculated as pairwise distances between samples of different subjects in the same group. Grouping was based on median splits of the improvement of symptom severity scores and BMI scores between data before (T1) and after weight rehabilitation (T2). BMI Groups with higher increments during inpatient treatment were defined as BMI increase > 2 and groups with lower increments during inpatient treatment were defined as BMI increase ≤ 2. Statistical tests were performed using the Mann–Whitney U test and Permutational Multivariate Analysis of Covariance (PERMANCOVA) to adjust for BMI as a covariate. Results in parentheses indicate non-significant results after adjustment for BMI using PERMANCOVA. A *p* < 0.05 was considered as statistically significant. * indicates a *p* < 0.05, ** indicates a *p* < 0.01, *** indicates a *p* < 0.001 and ns indicates not significant.

Considering the improvement in symptom severity scores between T1 and T2, similar patterns emerged (Table S8). After adjusting the data for BMI, no significant group differences remained between groups with higher and lower improvement in symptoms for anxiety and depression (Figure 4, Tables S9–S11).

### 3.4. Principal Coordinate Analysis and Distance-Based Redundancy Analysis

**Figure 5:**
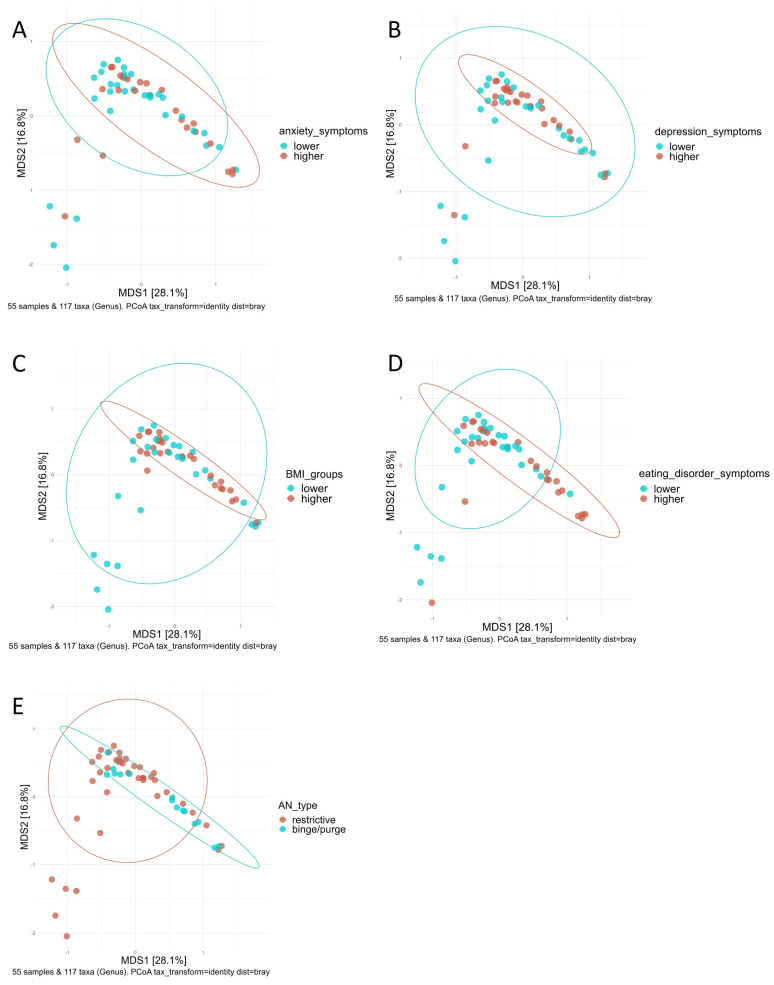
Principal Coordinate Analyses (PCoAs) based on Bray–Curtis distance in patients with anorexia nervosa (AN) in relation to body weight status (body mass index, BMI) and the severity of symptoms for eating disorder pathology, anxiety and depression before weight rehabilitation: PCoA colored according to groups with lower (blue) and higher (orange) severity of anxiety symptoms (**A**); PCoA colored according to groups with lower (blue) and higher (orange) severity of depression symptoms (**B**); PCoA colored according to lower (blue) and higher (orange) BMI groups. Lower BMI groups were defined as BMI ≤ 15.3. Higher BMI groups were defined as BMI > 15.3 (**C**); PCoA colored according to groups with lower (blue) and higher (orange) severity of eating disorder pathology symptoms (**D**); PCoA colored according to restrictive (orange) and binge/purge (blue) ANtype (**E**).

The visualization of Bray–Curtis distance by PCoA at T1 supported these findings (Figure 5). There were visible patterns for groups of AN type, BMI, and severity of depression, anxiety, and eating disorder symptoms.

**Figure 6:**
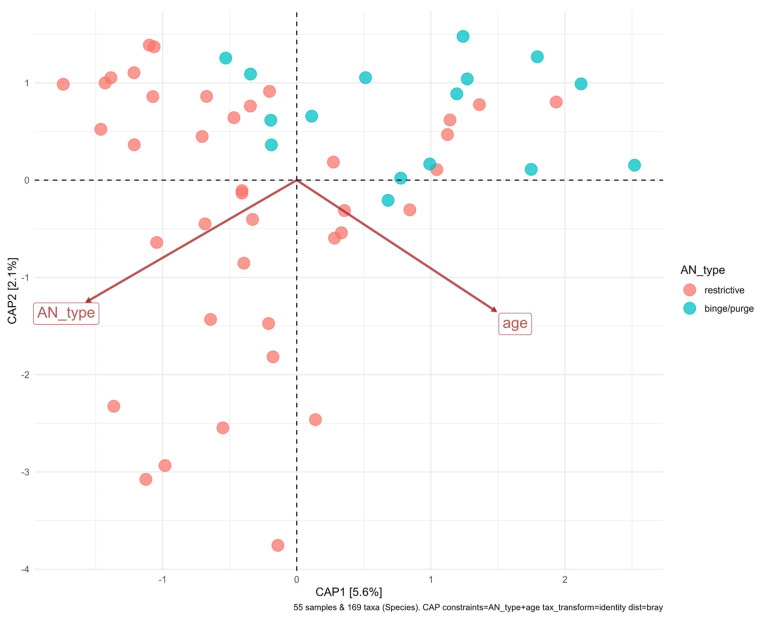
Distance-based Redundancy Analysis (db-RDA) plot showing the relationship of anorexia nervosa type (AN_type) and age with the microbial community structure: The analysis is based on the Bray–Curtis distance at baseline before weight rehabilitation. AN type (*p* = 0.017) and to a lesser extent age (*p* = 0.099, trend) were influential factors. Other variables (body mass index, macronutrient and energy intake, gastrointestinal symptoms as well as depression, anxiety, and eating disorder symptoms) had no significant effect on the microbiota. AN subtypes are colored in orange (restrictive type) and blue (binge/purge type).

In addition, db-RDA at T1 based on the Bray–Curtis distance was used to identify explanatory variables for the microbial community structure. As shown in Figure 6, the AN type was found to be a significant factor (*p* = 0.017). Furthermore, age also tended to be connected with the microbial community structure (*p* = 0.099). Other variables such as BMI, macronutrient and energy intake, gastrointestinal symptoms as well as symptoms for depression, anxiety, and eating disorder psychopathology, did not show a significant relationship with microbial composition. Thus, the same model remained as reported before [27] but took psychological variables into account too.

### 3.5. Microbial Composition

#### 3.5.1. Relationships between the Abundances of Taxa with Symptom Severity of Depression, Anxiety, and Eating Disorders before Adjusting for the BMI

The microbiota composition of patients with AN compared to normal-weight individuals and in the time course was previously reported by Mack et al. [27]. In these analyses, no correlation of taxa with symptom severity for depression and anxiety was found at T1. However, a correlation between the severity of eating disorder pathology and the relative and absolute abundance of *Bacteroides* (relative: *r*(53) = 0.43, *p* = 0.001, FDR = 0.06; absolute: *r*(53) = 0.38, *p* = 0.004, FDR = 0.1), *Erysipelotrichaceae* (relative: *r*(53) = - 0.47, *p* < 0.001, FDR = 0.04; absolute: *r*(53) = 0.45, *p* < 0.001, FDR = 0.07), *Eubacterium eligens* group (relative: *r*(53) = −0.39, *p* < 0.001, FDR = 0.109; absolute: *r*(53) = −0.4, *p* = 0.003, FDR = 0.1), *UCG 005* (relative: *r*(53) = −0.38, *p* < 0.001, FDR = 0.11; absolute: *r*(53) = −0.38, *p* = 0.004, FDR = 0.1) and *Coprococcus* (relative: *r*(53) = −0.39, *p* = 0.003, FDR = 0.11; absolute: *r*(53) = −0.38, *p* = 0.004, FDR = 0.1) was found. At T2, the absolute abundance of bacteria from the family *Ruminococcaceae* was significantly correlated with depression symptom severity (*r*(42) = 0.44, *p* = 0.003, FDR = 0.13).

Analyzing the relationship between changes in taxa and in symptom severity between T1 and T2, there were significant correlations between depression symptom severity and the relative abundance of the archeae *Euryarchaeota* (*r*(42) = 0.44, *p* = 0.004, FDR = 0.02), the absolute and relative abundance of the order *Gastranaerophilales* (relative: *r*(42) = 0.44, *p* = 0.003, FDR = 0.07; absolute: *r*(42) = 0.44, *p* = 0.003, FDR = 0.066) and the family *Methanobacteriaceae* (relative: *r*(42) = 0.45, *p* = 0.002, FDR = 0.07; absolute: *r*(42) = 0.46, *p* = 0.002, FDR = 0.07), the absolute abundance of the family *Erysipelotrichaceae* (*r*(42) = −0.41, *p* = 0.006, FDR = 0.09) as well as the relative abundance of the genera *Methanobrevibacter* (*r*(42) =−0.48, *p* = 0.002, FDR = 0.12) and bacteria of the *Clostridium innocuum* group (*r*(42) = −0.46, *p* = 0.002, FDR = 0.12). In addition, negative correlations emerged between the severity of anxiety symptoms and the absolute abundance of the family *Enterobacteriaceae* (*r*(42) = −0.4, *p* = 0.008, FDR = 0.17), the absolute and relative abundance of the family *Erysipelotrichaceae* (relative: *r*(42) = −0.45, *p* = 0.002, FDR = 0.11; absolute: *r*(42) = −0.45, *p* = 0.003, FDR = 0.12) and the genus *Turicibacter* (relative: *r*(42) = −0.48, *p* = 0.001, FDR = 0.09; absolute: *r*(42) = −0.49, *p* = 0.001, FDR = 0.1) as well as the relative abundance of bacteria of the *Clostridium innocuum* group (*r*(42) = −0.47, *p* = 0.001, FDR = 0.09). Correlations between the symptom severity of depression, anxiety, and eating disorders before and after weight rehabilitation and their changes during inpatient treatment according to additional questionnaires and the relative abundance of taxa on the family level are available in Table S12.

#### 3.5.2. Relationships between Abundances of Taxa with Symptom Severity of Depression, Anxiety and Eating Disorder Adjusted for the BMI

After adjusting for the BMI using linear models, no association remained between taxa and depression, anxiety, and eating disorder pathology scores at T1. At T2, the absolute abundance of bacteria of the phylum *Firmicutes* was significantly associated with anxiety symptom severity (β = 487, *SE* = 167, *t* = 2.91, *p* = 0.006, FDR = 0.05). At genus level, there was a significant association between depression severity at T2 and *Blautia* (relative: β = 0.0025, *SE* = 0.0007, *t* = 3.78, *p* <.001, FDR = 0.05; absolute: β = 127, *SE* = 29.6, *t* = 4.27, *p* < 0.001, FDR = 0.02), *Ruminococcus* (relative: β = 0.0038, *SE* = 0.0001, *t* = 3.95, *p* < 0.001, FDR = 0.05; absolute: β = 198, *SE* = 198, *t* = 4.09, *p* < 0.001, FDR = 0.02) and the absolute abundance of bacteria of the *Ruminococcus torques* group (β =18.6, *SE* = 6.49, *t* = 2.86, *p* = 0.007, FDR = 0.01).

Considering the relationships between changes in taxa at phylum, family, and genus levels and improvements in symptom severities, a significant association between the relative abundance of the genus *Turicibacter* and improvement in anxiety disorder was found (β = −842.16, *SE* = 394, *t* = −2.13, *p* = 0.04).

### 3.6. Relationship between SCFAs and Symptom Severity of Depression, Anxiety and Eating Disorder

#### 3.6.1. Relationships between the SCFAs with Symptom Severity of Depression, Anxiety, and Eating Disorder before Adjusting for the BMI

The comparison of SCFA concentrations and proportions between patients with AN and normal-weight individuals was published before by Mack et al. [27]. In this analysis, a negative association of valerate concentrations with the eating disorder symptom severity at T1 (*r*(53) = −0.33, *p* = 0.01) and of isovalerate concentrations with symptom severity of anxiety disorder at T2 (*r*(42) = 0.34, *p* = 0.03) was found. Considering the changes in SCFAs and symptom severity scores between T1 and T2, a negative correlation of acetate (*r*(42) = −0.33, *p* = 0.03) and propionate (*r*(43) = −0.32, *p* = 0.05) concentrations and improvement of depression severity emerged. Correlations between the symptom severity of depression, anxiety, and eating disorder before and after weight rehabilitation and their changes during inpatient treatment according to additional questionnaires and SCFAs are available in Table S13.

#### 3.6.2. Relationships between the SCFAs with Symptom Severity of Depression, Anxiety and Eating Disorder after Adjusting for the BMI

After adjusting the analysis for BMI using linear models, no relationship between SCFA concentrations and symptom severities remained at T1. At T2, isovalerate showed a relation to the symptom severity of anxiety disorder (*p* = 0.025). Looking at the changes in SCFAs and improvements in symptom severities between T1 and T2, propionate (β = −0.4369, *SE* = 0.1746, *t* = −2.5, *p* = 0.017) and acetate (β = −24.2, *SE* = 0.9454, *t* = −2.56, *p* = 0.019) are significantly associated with the improvement of depression severity.

## 4. Discussion

In this study, we investigated the relationship between the gastrointestinal microbiota and symptoms of depression, anxiety, and eating disorder pathology in patients with AN before and after weight restoration by reanalyzing the data of the MICROBIAN study [27]. The findings were controlled for the weight status.

Since inverse relationships between the BMI and symptoms of depression are well documented for underweight patients, an adjustment of the findings for the BMI is mandatory [25,26]. We found no significant differences in alpha and beta diversity measurements between groups of higher and lower symptom severity levels for depression and anxiety after adjusting for the BMI. This finding outlines the influence of weight and weight gain on the gastrointestinal microbiota and psychological parameters. To the best of our knowledge, the studies that reported a negative relationship between alpha diversity and depression symptoms [21] or significant correlations between specific bacteria or SCFAs and anxiety or depression [23] did not adjust for the BMI, and these observations may be due to differences of the weight status. Besides that, the results of this study approve indications of previous studies in patients with AN reporting BMI as a major factor influencing depression and anxiety as well as the gastrointestinal microbial community [23,24]. Regarding alpha diversity, our results share similarities with Schulz et al., who reported no associations with depression and anxiety scores in adolescent patients with AN before and after weight gain as well as the difference between these timepoints [22]. Regarding the relationship between anxiety and depression in general with the gastrointestinal microbiota, Simpson et al. summarized 24 human studies analyzing the microbiota in patients with depression and seven studies observing the microbiota in patients with anxiety [19]. In short, most studies reported no differences in alpha diversity measurements between patients with anxiety or depression compared to controls (*n* = 10). Only two studies observed a lower alpha diversity in patients with anxiety or depression, implying that most studies are in good agreement with our findings. With respect to beta diversity, current research is contradictory. According to Simpson et al., nine studies discovered differences in beta diversity in patients with anxiety or depression, whereas six studies did not found them [19]. Interestingly, Simpson et al. mentioned that only two studies deal with all relevant confounders, such as medication use, dietary intake, and BMI. As a consequence, they point out its relevance for the analysis and interpretation of further research [19]. Among these studies, Jackson et al. described associations of common diseases with the gut microbiota within 2737 individuals in the TwinsUK cohort. They found that models adjusted for BMI and age were highly correlated with unadjusted analysis, indicating that BMI and age have little influence on the relationship between microbiota and common diseases [60]. Also, Maes et al. did not find the effects of BMI, sex, age, and drug state in regression analysis with isometric log-ratio transformed the abundance of gut bacteria in patients with major depression disorder [61]. However, it needs to be mentioned that the mean BMI in both studies were in the slightly overweight range [60,61], thus these populations are not comparable with a malnourished, underweight population. Also, Rong et al. analyzed the relationship between BMI and gastrointestinal microbiota in patients with major depression disorder with a mean BMI of 21.5 [62]. They found a significant positive correlation of BMI with Shannon and Inverse Simpson indices as well as a negative correlation of BMI with the Gini coefficient, which measures the dissimilarity in relative abundances of microbiota [62]. These findings as well as our data link to the possible influence of the BMI on the gastrointestinal microbiota in context of depression and anxiety.

Possible mechanisms for the interactions between gut microbiota and psyche via the gut–brain axis are the synthesis of micronutrients, the regulation of inflammation, the production of neurotransmitters, and SCFAs by the members of the microbiota [63]. After inpatient treatment at T2, we found a positive association of the relative and absolute abundance of *Ruminococcus* and *Blautia* with depression severity even after adjusting for BMI. In contrast, Kleiman et al. reported a strong negative association between depression and eating disorder severity and *Ruminococcaceae* in patients with AN. However, it did not remain significant after the FDR correction [21]. In line with this, multiple studies in major depression disorder reported a reduction of *Ruminococcus* [61,64,65], but not all [66,67]. One explanation for contradictions with earlier findings regarding the extent of correlations between *Ruminococcus* and depression symptom severity could be the timepoint of analysis in our study. Kleiman et al. assessed the relationship between psychopathology scores and microbiota before weight gain [21]. Since our results only show a positive relationship at T2, this could be due to the normalization of eating habits as *Ruminococcus* is known for its role in gut health and its function in degrading polysaccharides [68]. There are also controversial outcomes about the connection between *Blautia* and depression in previous studies with normal-weight populations. Some of them observed a higher abundance of *Blautia* [64,66,69,70] and a positive correlation with depression [70], whereas others did not [71,72]. In addition, we found a significant positive association of the symptom severity of anxiety disorder with bacteria of the phylum *Firmicutes* at T2 after adjusting for BMI. Other analyses in patients with AN didn’t find a relationship between *Firmicutes* and anxiety but mentioned its positive correlation with BMI [73]. *Firmicutes* and their members, such as *Blautia* and *Ruminococcus*, are known to produce SCFAs by metabolization of dietary carbohydrates. Therefore, in this study, the positive associations between members of *Firmicutes* and depression and anxiety symptom severities at the end of inpatient treatment could be influenced by the normalization of dietary habits.

In this study, a higher reduction of depression severity scores during inpatient treatment was positively correlated with propionate and acetate levels. In rat models, multiple studies showed an antidepressant effect of sodium propionate administration [74,75]. Propionate is proposed to influence the neurotransmitter composition in the central nervous system. Low-dose sodium propionate administration led to a restoration of norepinephrine, dopamine, and gama-aminobutyric acid in rats exposed to chronic unpredictable mild stress, a model for depression. In line with this, a reduction in depression-like behavior was observed in these animals [74]. Furthermore, the administration of prebiotics in mice induced an increase of cecal acetate and propionate, which correlated with positive effects on depression- and anxiety-like behavior [76]. These findings point out the potential of propionate and acetate as a possible therapeutic approach for psychiatric disorders such as depression and anxiety, also when being a comorbidity of AN.

Besides questioning the relationship between the gastrointestinal microbiota and depression and anxiety in AN, this study demonstrates relationships between the gastrointestinal microbiota with the AN type, eating disorder psychopathology, and BMI. This implies that eating behavior and starvation together are the major influencing factors on the gastrointestinal microbial community in patients with AN. Possibly, existing relationships of depression and anxiety with the gastrointestinal microbiota in AN may be covered by these aforementioned strong factors. Our results regarding eating disorder pathology are consistent with previous findings. Castellini et al. reported a significant relationship between the gastrointestinal microbial community and eating disorder pathology in patients with AN after adjusting for BMI [24]. In addition, a negative correlation between alpha diversity and eating disorder pathology was discovered by Kleiman et al. [21]

This study has several strengths and limitations. Several questionnaires were used to assess depression, anxiety, and eating disorder pathology for each parameter. The analysis showed high correlations between those instruments and similar outcomes in relation to analysis considering the gastrointestinal microbiota (see methods). As all participants of the longitudinal arm were part of an inpatient treatment program for weight rehabilitation, a standardized diet was provided. In addition, complex analyses with adjustments for BMI instead of simple correlations were conducted to account for the weight status of these undernourished patients. Nevertheless, we are aware that this study can only reflect correlations and no causality. Furthermore, it is important to mention that the gut microbiota is influenced by many more factors than weight or weight gain. Besides, microbial and SCFA composition does not allow for insights into the functional capabilities of the gastrointestinal microbiota and the mechanism of the gut–brain axis through neuronal, immunological, and metabolic pathways. Consequently, this study offers insights into taxonomic composition rather than functional mechanisms in AN.

Recognizing the limitations of this study, future research could endeavor to investigate those functional mechanisms of the gut–brain axis in patients with AN and the role of malnutrition in this complex interplay. Insights in those aspects could help to improve the understanding of potential therapeutic approaches for AN involving gut microbiota modulation, such as the administration of pre-, pro-, and synbiotics. The modulation of the gastrointestinal microbiota is discussed for depression and anxiety disorders in humans [77,78,79]. However, practical applications for patients with AN cannot be derived from the current data available.

## 5. Conclusions

This paper highlights the importance of the BMI status in underweight when investigating the relationships between depression, anxiety, and gastrointestinal microbiota. Our findings suggest that if there is a relationship between depression and anxiety with the gastrointestinal microbiota in AN, it may be covered by the influence of eating pathology and malnutrition. Nevertheless, a relationship between *Ruminococcus* and *Blautia* as well as propionate and acetate with depression emerged independent from BMI. Consequently, further research about these relationships will be needed to evaluate the connection with depression and its relevance for adjunct depression treatment. The present findings contribute to our understanding of the complex interplay between physiological, psychological, and microbial factors in patients with AN.

## Figures and Tables

**Table 1:** Cut-off values used to group symptom severity of anxiety, depression, and eating disorders according to median splits before (T1) and after weight rehabilitation (T2).

	T1		T2	
Symptom Severity	Higher	Lower	Higher	Lower
Anxiety (GAD-7)	>11	≤11	>5	≤5
Depression (PHQ-9)	>14	≤14	>5	≤5
Eating disorder (EDI-II)	>82	≤82	>31	≤31

Generalized Anxiety Disorder Questionnaire (GAD-7), Patient Health Questionnaire 9 (PHQ-9), Eating Disorder Inventory (EDI-II).

**Table 2:** Values used to group the improvement in symptom severity of anxiety, depression, and eating disorders (∆T2 − T1) according to median splits.

Improvement of Symptom Severity (∆T2 − T1)	Higher	Lower
Anxiety (GAD-7)	≤−5	>−5
Depression (PHQ-9)	≤−8	>−8
Eating disorder (EDI-II)	≤−44	>−44

Generalized Anxiety Disorder Questionnaire (GAD-7), Patient Health Questionnaire 9 (PHQ-9), Eating Disorder Inventory (EDI-II).

**Table 3:** Characteristics of patients with AN before (T1) and after weight rehabilitation (T2). The differences between T1 and T2 were calculated for paired datasets (*n* = 44).

	T1(*n* = 55) Mean (SD) (Min–Max)	T2 (*n* = 44) Mean (SD) (Min–Max)	*p*-Value
Age	23.8 (6.8) years (min–max: 14–39)	23.1 (6.7) years (min–max: 14–39)	0.944
BMI	15.4 (1.4) (min–max: 11.6–17.7) kg/m^2^	17.7 (1.4) (min–max: 13.9–21.8) kg/m^2^	<0.001
Anxiety symptoms (GAD-7)	10.6 (4.7) (min–max: 2–21)	5.4 (4.0) (min–max: 0–17)	<0.001
Depression symptoms (PHQ-9)	14.1 (5.5) (min–max: 0–25)	5.6 (4.4) (min–max: 0–21)	<0.001
Eating disorder symptoms (EDI-II)	87.1 (38.6) (min–max: 20–194)	42.0 (33.4) (min–max: 6–167)	<0.001

The differences between T1 and T2 were calculated for paired datasets (*n* = 44). Body Mass Index (BMI), Generalized Anxiety Disorder Questionnaire (GAD-7), Patient Health Questionnaire 9 (PHQ-9), Eating Disorder Inventory (EDI-II).

## Data Availability

The v4 16S rDNA bacterial sequences used in this paper are found at the EMBL platform with accession No. PRJEB11199.

## Supplementary Materials

The following supporting information can be downloaded at: https://www.mdpi.com/article/10.3390/nu16060891/s1, Table S1: Mann–Whitney U test for differences in alpha diversity measurements between groups with higher and lower symptom severity of anxiety, depression, and eating disorder pathology and groups with higher and lower BMI before (T1) and after weight rehabilitation (T2). Table S2: Spearman correlations of symptoms for anxiety, depression, eating disorder pathology, and BMI with alpha diversity indices before (T1) and after weight rehabilitation (T2). Table S3: Mann–Whitney U test for differences in alpha diversity indices between groups with higher and lower improvement of anxiety, depression, and eating disorder pathology symptoms and groups with higher and lower increase of BMI during inpatient treatment. Table S4: Mann–Whitney-U test for differences in beta diversity measurements between groups with higher and lower symptom severity of anxiety, depression, and eating disorder pathology and groups with higher and lower BMI before (T1) and after weight rehabilitation (T2). Table S5: Beta diversity (Bray–Curtis index) in patients with Anorexia Nervosa in relation to body weight status and the severity of symptoms for eating disorder pathology, anxiety, and depression before (T1) and after weight rehabilitation (T2). Permutational Multivariate Analysis of Covariance (PERMANCOVA) was used to adjust for Body Mass Index (BMI). Table S6: Beta diversity (unweighted Unifrac distance) in patients with Anorexia Nervosa in relation to body weight status and the severity of symptoms for eating disorder pathology, anxiety, and depression before (T1) and after weight rehabilitation (T2). Permutational Multivariate Analysis of Covariance (PERMANCOVA) was used to adjust for Body Mass Index (BMI). Table S7: Beta diversity (weighted Unifrac distance) in patients with Anorexia Nervosa in relation to body weight status and the severity of symptoms for eating disorder pathology, anxiety, and depression before (T1) and after weight rehabilitation (T2) and their changes during inpatient treatment (longitudinal). Permutational Multivariate Analysis of Covariance (PERMANCOVA) was used to adjust for Body Mass Index (BMI). Table S8: Mann–Whitney-U test for differences in beta diversity measurements between groups with higher and lower improvement of anxiety, depression, and eating disorder pathology symptoms and groups with higher and lower increase of BMI during inpatient treatment. Table S9: Beta diversity (Bray–Curtis index) in patients with Anorexia Nervosa before (T1) and after weight rehabilitation (T2) in relation to the degree of improvement of body weight, symptoms for eating disorder pathology, anxiety, and depression during inpatient treatment. Permutational Multivariate Analysis of Covariance (PERMANCOVA) was used to adjust for Body Mass Index (BMI). Table S10: Beta diversity (unweighted Unifrac distance) in patients with Anorexia Nervosa before (T1) and after weight rehabilitation (T2) in relation to the degree of improvement of body weight, symptoms for eating disorder pathology, anxiety, and depression during inpatient treatment. Permutational Multivariate Analysis of Covariance (PERMANCOVA) was used to adjust for Body Mass Index (BMI). Table S11: Beta diversity (weighted Unifrac distance) before (T1) and after weight rehabilitation (T2) in patients with Anorexia Nervosa in relation to the degree of improvement of body weight, symptoms for eating disorder pathology, anxiety, and depression during inpatient treatment. Permutational Multivariate Analysis of Covariance (PERMANCOVA) was used to adjust for Body Mass Index (BMI). Table S12: Spearman correlation of additional questionnaires for symptom severity of eating disorder pathology, anxiety, and depression before (T1) and after weight rehabilitation (T2) and their changes during inpatient treatment (longitudinal) with the relative abundance of taxa on the family level. Table S13: Spearman correlation of additional questionnaires for symptom severity of eating disorder pathology, anxiety, and depression before (T1) and after weight rehabilitation (T2) and their changes during inpatient treatment (longitudinal) with Short Chain Fatty Acids (SCFA).
