# Supplementary material for: Gastrointestinal Microbiota & Symptoms of Depression and Anxiety in Anorexia Nervosa—A Re-Analysis of the MICROBIAN Longitudinal Study
Source: Nutrients. 2024 Mar 19;16(6):891. doi: 10.3390/nu16060891 (PMC10974745; doi:10.3390/nu16060891)
Supplement: Supplementary file 1 [file nutrients-16-00891-s001.zip › nutrients-2879957-supplementary.pdf]

Supplements

Gastrointestinal Microbiota & Symptoms of Depression and Anxiety in Anorexia Nervosa—A Re-Analysis of the MICROBIAN Longitudinal Study

Jasmin Ketel [1,2], Miquel Bosch-Bruguera [1,2], Greta Auchter [1,2], Ulrich Cuntz [3,4], Stephan Zipfel [1,2], Paul Enck [1,2] and Isabelle Mack [1,2,*]

[1] Department of Psychosomatic Medicine and Psychotherapy, University Hospital Tübingen, 72076 Tübingen, Germany; jasmin.ketel@med.uni-tuebingen.de (J.K.); miquel.bosch-bruguera@med.uni-tuebingen.de (M.B.-B.); greta.schmidt@eloquens.de (G.A.); stephan.zipfel@med.uni-tuebingen.de (S.Z.); paul.enck@proton.me (P.E.)

[2] Centre of Excellence for Eating Disorders (KOMET), 72076 Tübingen, Germany

[3] Klinik Roseneck, Center for Behavioral Medicine, 83209 Prien am Chiemsee, Germany; ucuntz@schoen-kliniken.de

[4] Forschungsprogramm für Psychotherapieevaluation im Komplexen Therapiesetting, Paracelsus Medical University (PMU), 5020 Salzburg, Austria

* Correspondence: isabelle.mack@uni-tuebingen.de

Table S1. Mann-Whitney U test for differences in alpha diversity measurements between groups with higher and lower symptom severity of anxiety, depression and eating disorder pathology and groups with higher and lower BMI before (T1) and after weight rehabilitation (T2).

Psyche variables	Shannon index								Chao1 index								Inverse Simpson index								Gini Simpson index							
	T1				T2				T1				T2				T1				T2				T1				T2			
	U	*Z*	*p*	*r*	*U*	*Z*	*p*	*r*	*U*	*Z*	*p*	*r*	*U*	*Z*	*p*	*r*	*U*	*Z*	*p*	*r*	*U*	*Z*	*p*	*r*	*U*	*Z*	*p*	*r*	*U*	*Z*	*p*	*r*
PHQ-9	327	-1.96	0.398	-0.11	174	-1.3	.193	-0.2	307	-1.18	.238	-0.16	172	-1.35	0.178	-0.2	338	-0.66	0.509	-0.09	190	-0.91	0.363	-0.14	338	-0.66	0.509	-0.09	190	-0.91	0.363	-0.14
BDI-II	244	-0.64	0.519	-0.09	142	-1.93	.054	-0.29	243	-0.66	.508	-0.09	144	-1.87	0.061	-0.28	245	-0.62	0.533	-0.08	156	-1.56	0.118	-0.24	245	-0.62	0.533	-0.08	156	-1.56	0.118	-0.24
BSI-D	311	-2.1	0.836	-0.03	216	-0.49	.622	-0.07	252	-1.28	.202	-0.17	247	-.21	0.831	-0.03	333	-0.17	0.864	-0.02	198	-0.92	0.359	-0.14	333	-0.17	0.864	-0.02	198	-0.92	0.359	-0.14
GAD-7	403	-0.51	0.608	-0.1	167	-1.32	.186	-0.2	425	-0.89	.373	-0.12	128	-1.52	0.129	-0.23	402	-0.5	0.619	-0.07	181	-0.97	0.33	-0.15	402	-0.5	0.619	-0.07	181	-0.97	0.33	-0.15
BSI-A	324	-0.85	0.396	-0.11	192	-0.98	.325	-0.15	326	-0.82	.412	-0.11	187	-1.11	0.266	-0.17	342	-0.55	0.586	-0.07	197	-0.86	0.388	-0.13	342	-0.55	0.586	-0.07	197	-0.86	0.388	-0.13
EDI-II	485	-1.79	0.073	-0.24	222	-0.45	.65	-0.07	527	-2.5	.012	-0.34	210	-0.74	0.459	-0.11	464	-1.44	0.151	-0.19	200	-0.97	0.334	-0.15	464	-1.44	0.151	-0.19	200	-0.97	0.334	-0.15
SIAB	381	-0.04	0.966	-0.01	198	-1.01	.312	-0.15	436	-0.97	.333	-0.13	222	-0.44	0.663	-0.07	449	-1.18	0.237	-0.16	185	-1.29	0.201	-0.19	449	-1.18	0.237	-0.16	185	-1.29	0.201	-0.19
BMI	313	-1.09	0.278	-0.15	282	-0.93	.354	-0.14	333	-0.74	.459	-0.1	250	-0.18	0.86	-0.03	310	-1.13	0.258	-0.15	306	-1.49	0.137	-0.22	310	-1.13	0.258	-0.15	306	-1.49	0.137	-0.22

Grouping was based on median splits of the symptom severity and BMI scores. Patient Health Questionnaire 9 (PHQ-9), Beck Depression Inventory II (BDI-II), Brief Symptom Inventory scale 4 (BSI-D), **Generalized** Anxiety Disorder Questionnaire (GAD-7), Brief Symptom Inventory scale 5 (BSI-A), Eating Disorder Inventory (EDI-II), Structured Interview for Anorexic and Bulimic Eating Disorders (SIAB), Body Mass Index (BMI).

Table S2. Spearman correlations of symptoms for anxiety, depression, eating disorder pathology and BMI with alpha diversity indices before (T1) and after weight rehabilitation (T2).

Psyche variables	Shannon index						Chao1 index						Inverse Simpson index						Gini Simpson index					
	T1			T2			T1			T2			T1			T2			T1			T2		
	S	*p*	*rho*	S	*p*	*rho*	S	*p*	*rho*	S	*p*	*rho*	S	*p*	*rho*	S	*p*	*rho*	S	*p*	*rho*	S	*p*	*rho*
PHQ-9	27407	0.935	0.01	11111	0.302	0.16	26544	0.758	0.04	11836	0.5	0.12	27200	0.892	0.02	9781	0.188	0.21	27200	0.892	0.02	9781	0.188	0.21
BDI-II	16822	0.855	0.03	9386	0.127	0.24	16470	0.75	0.05	10380	0.31	0.16	16714	0.822	0.03	9781	0.188	0.21	16714	0.822	0.03	9781	0.188	0.21
BSI-D	27260	0.904	0.02	12532	0.45	0.17	24930	0.465	0.1	13829	0.87	0.03	28591	0.82	-0.03	12461	0.431	0.12	28591	0.820	-0.03	12461	0.431	0.12
GAD-7	25709	0.599	0.07	10009	0.114	0.24	26245	0.7	0.05	10401	0.17	0.21	25757	0.607	0.07	10642	0.207	0.20	25757	0.607	0.07	10642	0.207	0.20
BSI-A	23593	0.278	0.15	13029	0.597	0.08	23936	0.32	0.14	12046	0.33	0.15	24415	0.386	0.12	13337	0.698	0.06	24415	0.386	0.12	13337	0.698	0.06
EDI-II	35169	0.047	-0.27	14535	0.876	-0.02	35773	-0.291	-0.29	12464	0.432	0.12	34421	0.075	-0.24	13984	0.926	0.01	34421	0.075	-0.24	13984	0.926	0.01
SIAB	34244	0.084	-0.24	14562	0.866	-0.03	37133	0.011	-0.34	15403	0.581	-0.09	33094	0.156	-0.19	14185	0.998	0.00	33094	0.156	-0.19	14185	0.998	0.00
BMI	27287	0.91	0.02	27287	0.91	0.02	28180	0.904	-0.02	28180	0.904	-0.02	26716	0.793	0.04	17980	0.08	-0.27	26716	0.793	0.04	17980	0.08	-0.27

Patient Health Questionnaire 9 (PHQ-9), Beck Depression Inventory II (BDI-II), Brief Symptom Inventory scale 4 (BSI-D), Generalized Anxiety Disorder Questionnaire (GAD-7), Brief Symptom Inventory scale 5 (BSI-A), Eating Disorder Inventory (EDI-II), Structured Interview for Anorexic and Bulimic Eating Disorders (SIAB), Body Mass Index (BMI).

Table S1. Mann-Whitney U test for differences in alpha diversity indices between groups with higher and lower improvement of anxiety, depression and eating disorder pathology symptoms and groups with higher and lower increase of BMI during inpatient treatment.

Psyche variables	Shannon index								Chao1 index								Inverse Simpson index								Gini Simpson index							
	T1				T2				T1				T2				T1				T2				T1				T2			
	U	*Z*	*p*	*r*	*U*	*Z*	*p*	*r*	*U*	*Z*	*p*	*r*	*U*	*Z*	*p*	*r*	*U*	*Z*	*p*	*r*	*U*	*Z*	*p*	*r*	*U*	*Z*	*p*	*r*	*U*	*Z*	*p*	*r*
PHQ-9	209	-0.28	0.778	-0.04	224	-0.61	0.951	-0.01	214	-0.15	0.881	-0.02	224	-0.06	0.951	-0.01	204	-0.47	0.685	-0.06	226	-0.11	0.912	-0.02	204	-0.47	0.685	-0.06	226	-0.11	0.912	-0.02
BDI-II	140	-0.41	0.684	-0.05	140	-0.41	0.684	-0.06	152	0	1	0	140	-0.41	0.684	-0.06	140	-0.41	0.684	-0.06	137	-0.51	0.613	-0.08	140	-0.41	0.684	-0.06	137	-0.51	0.613	-0.08
BSI-D	207	-0.76	0.448	-0.1	218	-0.5	0.616	-0.08	251	-0.25	0.804	-0.03	218	-0.5	0.616	-0.08	212	-0.64	0.521	-0.10	194	-1.07	0.287	-0.16	212	-0.64	0.521	-0.10	194	-1.07	0.287	-0.16
GAD-7	251	-0.47	0.639	-0.06	267	-0.86	0.392	-0.13	212	-0.44	0.662	-0.06	267	-0.86	0.392	-0.13	244	-0.3	0.764	-0.05	265	-0.81	0.420	-0.12	244	-0.3	0.764	-0.05	265	-0.81	0.420	-0.12
BSI-A	303	-1.47	0.142	-0.2	272	-0.74	0.462	-0.11	289	-0.14	0.253	-0.15	272	-0.74	0.462	-0.11	300	-1.4	0.162	-0.21	272	-0.74	0.462	-0.11	300	-1.4	0.162	-0.21	272	-0.74	0.462	-0.11
EDI-II	217	-0.57	0.569	-0.08	264	-0.5	0.617	-0.08	190	-1.2	0.231	-0.16	264	-0.5	0.617	-0.08	217	-0.57	0.569	-0.09	273	-0.71	0.478	-0.11	217	-0.57	0.569	-0.09	273	-0.71	0.478	-0.11
SIAB	194	-1.11	0.268	-0.15	216	-0.59	0.553	-0.1	164	-1.81	0.07	-0.24	216	-0.59	0.553	-0.09	197	-1.04	0.3	-0.16	221	-0.48	0.634	-0.07	197	-1.04	0.3	-0.16	221	-0.48	0.634	-0.07
BMI	153	-2.08	0.038	0.28	192	-1.14	0.252	-0.17	216	-0.59	0.557	-0.08	192	-1.14	0.252	-0.17	339	-2.3	0.022	-0.35	297	-2.3	0.198	-0.35	339	-2.3	0.022	-0.35	297	-2.3	0.198	-0.35

Grouping was based on median splits of the increase in symptom severity and BMI scores between T1 and T2. Patient Health Questionnaire 9 (PHQ-9), Beck Depression Inventory II (BDI-II), Brief Symptom Inventory scale 4 (BSI-D), Generalized Anxiety Disorder Questionnaire (GAD-7), Brief Symptom Inventory scale 5 (BSI-A), Eating Disorder Inventory (EDI-II), Structured Interview for Anorexic and Bulimic Eating Disorders (SIAB), Body Mass Index (BMI)

Table S2. Mann-Whitney-U test for differences in beta diversity measurements between groups with higher and lower symptom severity of anxiety, depression and eating disorder pathology and groups with higher and lower BMI before (T1) and after weight rehabilitation (T2).

Psyche variables	Bray-Curtis index								Unweighted Unifrac distance								Weighted Unifrac distance							
	T1				T2				T1				T2				T1				T2			
	U	*Z*	*p*	*r*	*U*	*Z*	*p*	*r*	*U*	*Z*	*p*	*r*	*U*	*Z*	*p*	*r*	*U*	*Z*	*p*	*r*	*U*	*Z*	*p*	*r*
PHQ-9	43509	-7.14	< 0.001	-0.96	13581	-6.59	< 0.001	-0.99	61050	-1.86	0.063	-0.25	15561	-6.05	< 0.001	-0.91	48866	-4.94	< 0.001	-0.67	15182	-6.34	< 0.001	-0.96
BDI-II	20726	-7.04	< 0.001	-0.95	17711	-3.17	< 0.01	-0.48	29196	-3.11	< 0.01	-0.42	16236	-4.36	< 0.001	-0.66	23293	-5.34	< 0.001	-0.72	18411	-2.6	< 0.01	-0.39
BSI-D	37032	-2.15	< 0.05	-0.29	20944	-3.31	< 0.001	-0.5	37171	-2.06	< 0.05	-0.28	23554	-1.48	0.14	-0.22	37564	-1.78	< 0.001	-0.24	20673	-3.5	< 0.001	-0.53
GAD-7	59726	-1.58	0.115	-0.21	13609	-5.38	< 0.001	-0.81	78108	-3.37	< 0.001	-0.45	18503	-2.76	< 0.01	-0.42	59263	-1.74	0.081	-0.23	13501	-5.48	< 0.001	-0.83
BSI-A	69877	-1.64	0.1	-0.22	23586	-0.91	0.365	-0.14	66533	-0.45	0.65	-0.06	23026	-1.3	0.192	-0.2	75439	-3.6	< 0.001	-0.49	24679	-0.13	0.897	-0.02
EDI-II	62430	-1.38	0.169	-0.18	34878	-4.39	< 0.001	-0.66	97615	-8.21	< 0.001	-1.1	33926	-3.51	< 0.001	-0.53	48028	-6.45	< 0.001	-0.87	36842	-7.08	< 0.001	-1.07
SIAB	50063	-5.73	< 0.001	-0.77	19417	-5	< 0.001	-0.75	71458	-3.37	0.07	-0.45	26982	-0.29	0.771	-0.04	48028	-6.45	< 0.001	-0.87	19717	-4.78	< 0.001	-0.72
BMI	88383	-6.83	< 0.001	-0.92	35512	-4.95	< 0.001	-0.75	65616	-0.25	0.799	-0.03	21291	-3.76	< 0.001	-0.57	95252	-8.21	< 0.001	-1.12	43063	-8.21	< 0.001	-1.24

Grouping was based on median splits of the symptom severity and BMI scores. Patient Health Questionnaire 9 (PHQ-9), Beck Depression Inventory II (BDI-II), Brief Symptom Inventory scale 4 (BSI-D), Generalized Anxiety Disorder Questionnaire (GAD-7), Brief Symptom Inventory scale 5 (BSI-A), Eating Disorder Inventory (EDI-II), Structured Interview for Anorexic and Bulimic Eating Disorders (SIAB), Body Mass Index (BMI).

Table S5. Beta diversity (Bray-Curtis index) in patients with Anorexia Nervosa in relation to body weight status and the severity of symptoms for eating disorder pathology, anxiety, and depression before (T1) and after weight rehabilitation (T2). Permutational Multivariate Analysis of Covariance (PERMANCOVA) was used to adjust for Body Mass Index (BMI).

	T1					T2				
Depression	Df	SumOfSqs	R2	F	Pr(>F)	Df	SumOfSqs	R2	F	Pr(>F)
PHQ-9	1	0.15	0.0164	0.91	0.48	1	0.15	0.02402	1.05	0.35
BMI	1	0.48	0.0512	2.85	0.01	1	0.40	0.06295	2.76	0.01
Residual	52	8.6845	0.9325			40	5.7594	0.91303		
Total	54	9.3133	1			42	6.3081	1		
BDI-II	1	0.1691	0.0211	0.994	0.381	1	0.1256	0.01992	0.8453	0.553
BMI	1	0.3586	0.04476	2.1082	0.045	1	0.3847	0.06101	2.5889	0.005
Residual	44	7.4836	0.93414			39	5.7956	0.91907		
Total	46	8.0113	1			41	6.3059	1		
BSI-D	1	0.0819	0.00879	0.488	0.928	1	0.1144	0.01776	0.785	0.622
BMI	1	0.5076	0.0545	3.0257	0.006	1	0.3512	0.05452	2.4093	0.018
Residual	52	8.7238	0.9367			41	5.9758	0.92772		
Total	54	9.3133	1			43	6.4414	1		
Anxiety disorder										
GAD-7	1	0.1958	0.02102	1.1806	0.284	1	0.1577	0.02501	1.0816	0.341
BMI	1	0.4946	0.05311	2.9827	0.009	1	0.3165	0.05018	2.1703	0.019
Residual	52	8.6229	0.92587			40	5.8338	0.92481		
Total	54	9.3133	1			42	6.3081	1		
BDI-A	1	0.0889	0.00955	0.5306	0.885	1	0.1276	0.01981	0.8815	0.517
BMI	1	0.5098	0.05474	3.0418	0.002	1	0.3785	0.05877	2.615	0.006
Residual	52	8.7146	0.93572			41	5.9352	0.92142		
Total	54	9.3133	1			43	6.4414	1		
Eating disorder										
EDI-II	1	0.4625	0.04966	2.827	0.01	1	0.1538	0.02388	1.0629	0.355
BMI	1	0.3437	0.03691	2.1012	0.041	1	0.3546	0.05505	2.4502	0.013
Residual	52	8.5071	0.91343			41	5.933	0.92108		
Total	54	9.3133	1			43	6.4414	1		
SIAB	1	0.6963	0.07476	4.3442	0.002	1	0.149	0.02313	1.0284	0.396
BMI	1	0.2825	0.03034	1.7628	0.092	1	0.3526	0.05475	2.4341	0.022
Residual	52	8.3345	0.8949			41	5.9398	0.92212		
Total	54	9.3133	1			43	6.4414	1		

Patient Health Questionnaire 9 (PHQ-9), Beck Depression Inventory II (BDI-II), Brief Symptom Inventory scale 4 (BSI-D), Generalized Anxiety Disorder Questionnaire (GAD-7), Brief Symptom Inventory scale 5 (BSI-A), Eating Disorder Inventory (EDI-II), Structured Interview for Anorexic and Bulimic Eating Disorders (SIAB), Body Mass Index (BMI).

Table S6. Beta diversity (unweighted Unifrac distance) in patients with Anorexia Nervosa in relation to body weight status and the severity of symptoms for eating disorder pathology, anxiety, and depression before (T1) and after weight rehabilitation (T2). Permutational Multivariate Analysis of Covariance (PERMANCOVA) was used to adjust for Body Mass Index (BMI).

	T1					T2				
Depression	Df	SumOfSqs	R2	F	Pr(>F)	Df	SumOfSqs	R2	F	Pr(>F)
PHQ-9	1	0.1840	0.0268	1.4617	0.126	1	0.13	0.02927	1.2671	0.173
BMI	1	0.1381	0.0201	1.0969	0.301	1	0.20	0.04676	2.0243	0.015
Residual	52	6.5474	0.9531			40	3.9831	0.92397		
Total	54	6.8696	1.0000			42	4.3109	1		
BDI-II	1	0.1668	0.02725	1.266	0.179	1	0.1144	0.02674	1.1158	0.292
BMI	1	0.1579	0.02579	1.1985	0.208	1	0.1658	0.03874	1.6167	0.075
Residual	44	5.7963	0.94696			39	3.9986	0.93452		
Total	46	6.1209	1			41	4.2788	1		
BSI-D	1	0.1116	0.01625	0.8763	0.515	1	0.0882	0.01995	0.8638	0.601
BMI	1	0.134	0.0195	1.0517	0.322	1	0.1464	0.03311	1.4337	0.123
Residual	52	6.6240	0.96425			41	4.1879	0.94694		
Total	54	6.8696	1			43	4.4226	1		
Anxiety disorder										
GAD-7	1	0.1316	0.01916	1.0362	0.312	1	0.1138	0.0264	1.1298	0.249
BMI	1	0.1314	0.01913	1.0342	0.363	1	0.1675	0.03886	1.6629	0.059
Residual	52	6.6065	0.96171			40	4.0295	0.93474		
Total	54	6.8696	1			42	4.3109	1		
BDI-A	1	0.1141	0.01661	0.8963	0.502	1	0.1047	0.02367	1.0315	0.369
BMI	1	0.1365	0.01987	1.0725	0.332	1	0.1571	0.03551	1.5476	0.078
Residual	52	6.619	0.96352			41	4.1609	0.94082		
Total	54	6.8696	1			43	4.4226	1		
Eating disorder										
EDI-II	1	0.2657	0.03867	2.1239	0.027	1	0.1359	0.03072	1.3451	0.129
BMI	1	0.0997	0.01451	0.7969	0.602	1	0.1455	0.0329	1.4405	0.099
Residual	52	6.5042	0.94682			41	4.1412	0.93638		
Total	54	6.8696	1			43	4.4226	1		
SIAB	1	0.3042	0.04429	2.4525	0.014	1	0.0583	0.01318	0.5672	0.956
BMI	1	0.115	0.01674	0.927	0.449	1	0.1514	0.03423	1.4731	0.091
Residual	52	6.4504	0.93897			41	4.2129	0.9526		
Total	54	6.8696	1			43	4.4226	1		

Patient Health Questionnaire 9 (PHQ-9), Beck Depression Inventory II (BDI-II), Brief Symptom Inventory scale 4 (BSI-D), Generalized Anxiety Disorder Questionnaire (GAD-7), Brief Symptom Inventory scale 5 (BSI-A), Eating Disorder Inventory (EDI-II), Structured Interview for Anorexic and Bulimic Eating Disorders (SIAB), Body Mass Index (BMI).

Table S7. Beta diversity (weighted Unifrac distance) in patients with Anorexia Nervosa in relation to body weight status and the severity of symptoms for eating disorder pathology, anxiety, and depression before (T1) and after weight rehabilitation (T2) and their changes during inpatient treatment (longitudinal). Permutational Multivariate Analysis of Covariance (PERMANCOVA) was used to adjust for Body Mass Index (BMI).

	T1					T2				
Depression	Df	SumOfSqs	R2	F	Pr(>F)	Df	SumOfSqs	R2	F	Pr(>F)
PHQ-9	1	0.05	0.02302	1.2662	0.218	1	0.04	0.02988	1.3470	0.216
BMI	1	0.07	0.03171	1.7445	0.118	1	0.12	0.08279	3.7323	0.002
Residual	52	2.0731	0.94527			40	1.2542	0.88733		
Total	54	2.1932	1			42	1.4135	1		
BDI-II	1	0.04653	0.02482	1.1466	0.274	1	0.04219	0.02984	1.3048	0.224
BMI	1	0.04268	0.02276	1.0516	0.337	1	0.11057	0.07821	3.4198	0.012
Residual	44	1.7857	0.95242			39	1.2609	0.89194		
Total	46	1.8749	1			41	1.4137	1		
BSI-D	1	0.01488	0.00678	0.3675	0.951	1	0.0341	0.02359	1.0662	0.331
BMI	1	0.07332	0.03343	1.8113	0.103	1	0.0999	0.06912	3.1233	0.012
Residual	52	2.1050	0.95979			41	1.3112	1		
Total	54	2.1932	1			43	1.44513	1		
Anxiety disorder										
GAD-7	1	0.02933	0.01337	0.729	0.574	1	0.03386	0.02395	1.0594	0.314
BMI	1	0.07192	0.03279	1.7879	0.109	1	0.10133	0.07169	3.1707	0.007
Residual	52	2.09192	0.95383			40	1.27831	0.90436		
Total	54	2.19317	1			42	1.4135	1		
BDI-A	1	0.02671	0.01218	0.6642	0.688	1	0.05063	0.03503	1.6212	0.119
BMI	1	0.07552	0.03444	1.8782	0.092	1	0.11412	0.07897	3.6543	0.005
Residual	52	2.09094	0.95339			41	1.28039	0.886		
Total	54	2.19317	1			43	1.44513	1		
Eating disorder										
EDI-II	1	0.09817	0.04476	2.505	0.04	1	0.06994	0.0484	2.2485	0.04
BMI	1	0.057	0.02599	1.4543	0.176	1	0.09994	0.06915	3.213	0.01
Residual	52	2.038	0.92925			41	1.27526	0.88245		
Total	54	2.19317	1			43	1.44513	1		
SIAB	1	0.07594	0.03462	1.9106	0.08	1	0.05082	0.03517	1.619	0.119
BMI	1	0.05054	0.02304	1.2716	0.225	1	0.10729	0.07424	3.4178	0.006
Residual	52	2.0667	0.94233			41	1.28702	0.89059		
Total	54	2.19317	1			43	1.44513	1		

Patient Health Questionnaire 9 (PHQ-9), Beck Depression Inventory II (BDI-II), Brief Symptom Inventory scale 4 (BSI-D), Generalized Anxiety Disorder Questionnaire (GAD-7), Brief Symptom Inventory scale 5 (BSI-A), Eating Disorder Inventory (EDI-II), Structured Interview for Anorexic and Bulimic Eating Disorders (SIAB), Body Mass Index (BMI).

Table S8. Mann-Whitney-U test for differences in beta diversity measurements between groups with higher and lower improvement of anxiety, depression and eating disorder pathology symptoms and groups with higher and lower increase of BMI during inpatient treatment.

Psyche variables	Bray-Curtis index								Unweighted Unifrac distance								Weighted Unifrac distance							
	T1				T2				T1				T2				T1				T2			
	U	*Z*	*p*	*r*	*U*	*Z*	*p*	*r*	*U*	*Z*	*p*	*r*	*U*	*Z*	*p*	*r*	*U*	*Z*	*p*	*r*	*U*	*Z*	*p*	*r*
PHQ-9	19227	-2.2	< 0.05	-0.3	18412	-2.83	< 0.001	-0.43	24364	-1.74	0.083	-0.23	25581	-2.67	< 0.01	-0.4	17942	-3.19	< 0.01	-0.43	16941	-1.77	0.076	0.27
BDI-II	7654	-3.9	< 0.001	-0.52	7732	-3.77	< 0.001	-0.57	10083	-0.45	0.651	-0.06	11444	-1.47	0.143	-0.22	7655	-3.88	< 0.001	-0.52	7101	-2.95	< 0.01	-0.44
BSI-D	27471	-0.88	0.381	-0.12	22321	-2.73	< 0.01	-0.41	27290	-0.75	0.454	-0.1	26279	-0.04	0.967	-0.01	28128	-1.34	0.182	-0.18	21184	-3.52	< 0.001	-0.53
GAD-7	23571	-0.51	0.609	-0.09	20162	-3.06	< 0.01	-0.46	23406	-0.63	0.526	-0.09	24680	-0.32	0.751	-0.05	23682	-0.43	0.668	-0.06	19877	-3.27	< 0.01	-0.49
BSI-A	26196	-0.02	0.987	0	25853	-0.26	0.798	-0.04	21584	-3.24	< 0.01	-0.44	19231	-3.29	< 0.001	-0.5	28585	-1.66	0.098	-0.22	26553	-0.23	0.816	-0.04
EDI-II	16360	-6.18	< 0.001	-0.83	14888	-8.21	< 0.001	-1.24	31884	-3.63	< 0.001	-0.49	32226	-3.86	< 0.001	-0.58	16049	-6.43	< 0.001	-0.87	16000	-6.5	< 0.001	-0.98
SIAB	18467	-4.41	< 0.001	-0.66	16244	-6.27	< 0.001	-0.95	36139	-5.48	< 0.001	-0.83	38862	-8.21	< 0.001	-1.24	16660	-5.94	< 0.001	-0.9	15593	-6.79	< 0.001	-1.02
BMI	18711	-5.48	< 0.001	-0.74	27329	-0.53	0.594	-0.08	21019	-3.87	< 0.001	-0.52	21765	-3.35	< 0.001	-0.5	20317	-2.48	< 0.05	-0.33	30975	-3.08	< 0.01	-0.46

Grouping was based on median splits of the increase in symptom severity and BMI scores between T1 and T2. Patient Health Questionnaire 9 (PHQ-9), Beck Depression Inventory II (BDI-II), Brief Symptom Inventory scale 4 (BSI-D), Generalized Anxiety Disorder Questionnaire (GAD-7), Brief Symptom Inventory scale 5 (BSI-A), Eating Disorder Inventory (EDI-II), Structured Interview for Anorexic and Bulimic Eating Disorders (SIAB), Body Mass Index (BMI).

Table S9. Beta diversity (Bray-Curtis index) in patients with Anorexia Nervosa before (T1) and after weight rehabilitation (T2) in relation to the degree of improvement of body weight, symptoms for eating disorder pathology, anxiety and depression during inpatient treatment. Permutational Multivariate Analysis of Covariance (PERMANCOVA) was used to adjust for Body Mass Index (BMI).

	T1					T2				
Depression	Df	SumOfSqs	R2	F	Pr(>F)	Df	SumOfSqs	R2	F	Pr(>F)
PHQ-9	1	0.10	0.01386	0.6	0.82	1	0.13	0.02106	0.9	0.49
BMI	1	0.42	0.06004	2.59	0.02	1	0.29	0.04651	2.	0.04
Residual	40	6.5475	0.92610			40	5.8818	0.93243		
Total	42	7.0700	1			42	6.3081	1		
BDI-II	1	0.1415	0.02338	0.8045	0.564	1	0.1517	0.02855	0.9856	0.422
BMI	1	0.2814	0.0465	1.5996	0.134	1	0.237	0.0446	1.5398	0.132
Residual	32	5.6300	0.93012			32	4.9263	0.92685		
Total	34	6.0529	1			34	5.3151	1		
BSI-D	1	0.1709	0.02374	1.0648	0.35	1	0.1233	0.01914	0.8468	0.579
BMI	1	0.4489	0.06233	2.7964	0.008	1	0.3485	0.05411	2.3937	0.017
Residual	41	6.5819	0.91393			41	5.9696	0.92675		
Total	43	7.2018	1			43	6.4414	1		
Anxiety disorder										
GAD-7	1	0.1683	0.0238	1.0451	0.365	1	0.1137	0.01802	0.7779	0.632
BMI	1	0.4607	0.06517	2.8612	0.006	1	0.3485	0.05525	2.3846	0.018
Residual	40	6.441	0.91103			40	5.8459	0.92673		
Total	42	7.07	1			42	6.3081	1		
BDI-A	1	0.1684	0.02338	1.0474	0.353	1	0.1771	0.0275	1.2277	0.252
BMI	1	0.4418	0.06134	2.7477	0.013	1	0.3497	0.05429	2.424	0.015
Residual	41	6.5916	0.91528			41	5.9146	0.91822		
Total	43	7.2018	1			43	6.4414	1		
Eating disorder										
EDI-II	1	0.2667	0.03703	1.6408	0.096	1	0.2763	0.04289	1.9128	0.04
BMI	1	0.2707	0.03759	1.6655	0.088	1	0.2434	0.03779	1.6853	0.068
Residual	41	6.6643	0.92538			41	5.9217	0.91932		
Total	43	7.2018	1			43	6.4414	1		
SIAB	1	0.5037	0.06995	3.2048	0.002	1	0.5206	0.08081	3.7496	0.003
BMI	1	0.2535	0.0352	1.6126	0.119	1	0.2288	0.03552	1.648	0.085
Residual	41	6.4445	0.89486			41	5.692	0.88367		
Total	43	7.2018	1			43	6.4414	1		

Patient Health Questionnaire 9 (PHQ-9), Beck Depression Inventory II (BDI-II), Brief Symptom Inventory scale 4 (BSI-D), Generalized Anxiety Disorder Questionnaire (GAD-7), Brief Symptom Inventory scale 5 (BSI-A), Eating Disorder Inventory (EDI-II), Structured Interview for Anorexic and Bulimic Eating Disorders (SIAB), Body Mass Index (BMI).

Table S10. Beta diversity (unweighted Unifrac distance) in patients with Anorexia Nervosa before (T1) and after weight rehabilitation (T2) in relation to the degree of improvement of body weight, symptoms for eating disorder pathology, anxiety, and depression during inpatient treatment. Permutational Multivariate Analysis of Covariance (PERMANCOVA) was used to adjust for Body Mass Index (BMI).

	T1					T2				
Depression	Df	SumOfSqs	R2	F	Pr(>F)	Df	SumOfSqs	R2	F	Pr(>F)
PHQ-9	1	0.12	0.0228	0.9566	0.389	1	0.11	0.02656	1.1264	0.264
BMI	1	0.13	0.0253	1.0620	0.311	1	0.13	0.03037	1.2880	0.175
Residual	40	5.0077	0.9520			40	4.0655	0.94308		
Total	42	5.2605	1			42	4.3109	1		
BDI-II	1	0.104	0.02281	0.7683	0.667	1	0.1209	0.03278	1.1265	0.28
BMI	1	0.1232	0.02702	0.9101	0.455	1	0.1335	0.0362	1.2444	0.221
Residual	32	4.3328	0.95017			32	3.4332	0.93102		
Total	34	4.5600	1			34	3.6876	1		
BSI-D	1	0.0774	0.01442	0.6144	0.886	1	0.0907	0.0205	0.8876	0.563
BMI	1	0.1241	0.02311	0.9844	0.394	1	0.1437	0.0325	1.4069	0.12
Residual	41	5.1673	0.96247			41	4.1882	0.947		
Total	43	5.3688	1			43	4.4226	1		
Anxiety disorder										
GAD-7	1	0.1668	0.03171	1.3448	0.156	1	0.1027	0.02381	1.0097	0.414
BMI	1	0.1327	0.02523	1.0703	0.295	1	0.1413	0.03277	1.3894	0.136
Residual	40	4.9609	0.94306			40	4.0669	0.94342		
Total	42	5.2605	1			42	4.3109	1		
BDI-A	1	0.0954	0.01778	0.7613	0.687	1	0.1452	0.03283	1.438	0.1
BMI	1	0.1335	0.02486	1.0648	0.314	1	0.1374	0.03106	1.3605	0.132
Residual	41	5.1399	0.95736			41	4.14	0.9361		
Total	43	5.3688	1			43	4.4226	1		
Eating disorder										
EDI-II	1	0.1409	0.02625	1.1234	0.294	1	0.152	0.03437	1.4925	0.078
BMI	1	0.0847	0.01578	0.6755	0.811	1	0.0955	0.0216	0.9381	0.46
Residual	41	5.1431	0.95797			41	4.1751	0.94403		
Total	43	5.3688	1			43	4.4226	1		
SIAB	1	0.2314	0.0431	1.8811	0.045	1	0.2617	0.05918	2.6429	0.004
BMI	1	0.0938	0.01748	0.7628	0.683	1	0.1009	0.02281	1.0189	0.392
Residual	41	5.0435	0.93942			41	4.06	0.91801		
Total	43	5.3688	1			43	4.4226	1		

Patient Health Questionnaire 9 (PHQ-9), Beck Depression Inventory II (BDI-II), Brief Symptom Inventory scale 4 (BSI-D), Generalized Anxiety Disorder Questionnaire (GAD-7), Brief Symptom Inventory scale 5 (BSI-A), Eating Disorder Inventory (EDI-II), Structured Interview for Anorexic and Bulimic Eating Disorders (SIAB), Body Mass Index (BMI).

Table S11. Beta diversity (weighted Unifrac distance) before (T1) and after weight rehabilitation (T2) in patients with Anorexia Nervosa in relation to the degree of improvement of body weight, symptoms for eating disorder pathology, anxiety, and depression during inpatient treatment. Permutational Multivariate Analysis of Covariance (PERMANCOVA) was used to adjust for Body Mass Index (BMI).

	T1					T2				
Depression	Df	SumOfSqs	R2	F	Pr(>F)	Df	SumOfSqs	R2	F	Pr(>F)
PHQ-9	1	0.03	0.01622	0.6884	0.66	1	0.03	0.01925	0.8316	0.548
BMI	1	0.07	0.04142	1.7582	0.136	1	0.08	0.05486	2.3702	0.034
Residual	40	1.5828	0.94236			40	1.3087	0.92589		
Total	42	1.6796	1			42	1.4135	1		
BDI-II	1	0.03171	0.0224	0.7558	0.539	1	0.05002	0.0415	1.4618	0.158
BMI	1	0.04126	0.02915	0.9834	0.361	1	0.0603	0.05003	1.7622	0.108
Residual	32	1.3427	0.94845			32	1.0950	0.90847		
Total	34	1.4157	1			34	1.2054	1		
BSI-D	1	0.04125	0.02423	1.0652	0.329	1	0.04263	0.0295	1.3339	0.205
BMI	1	0.0737	0.04328	1.9027	0.078	1	0.0922	0.06383	2.8864	0.018
Residual	41	1.5879	1			41	1.3103	1		
Total	43	1.70286	1			43	1.44513	1		
Anxiety disorder										
GAD-7	1	0.03203	0.01907	0.8156	0.524	1	0.02318	0.0164	0.7138	0.652
BMI	1	0.07679	0.04572	1.9555	0.09	1	0.09163	0.06482	2.8222	0.013
Residual	40	1.5708	0.93521			40	1.2987	0.91878		
Total	42	1.67962	1			42	1.4135	1		
BDI-A	1	0.04337	0.02547	1.1226	0.314	1	0.02316	0.01602	0.7168	0.643
BMI	1	0.07551	0.04434	1.9546	0.08	1	0.09737	0.06738	3.0139	0.015
Residual	41	1.58398	0.93019			41	1.3246	0.9166		
Total	43	1.70286	1			43	1.44513	1		
Eating disorder										
EDI-II	1	0.05844	0.03432	1.4945	0.169	1	0.0437	0.03024	1.3629	0.187
BMI	1	0.04105	0.02411	1.0498	0.332	1	0.08681	0.06007	2.7074	0.023
Residual	41	1.60336	0.94157			41	1.31462	0.90969		
Total	43	1.70286	1			43	1.44513	1		
SIAB	1	0.07412	0.04353	1.9238	0.073	1	0.08252	0.0571	2.63	0.021
BMI	1	0.04901	0.02878	1.272	0.21	1	0.07612	0.05267	2.4259	0.025
Residual	41	1.57973	0.92769			41	1.28649	0.89022		
Total	43	1.70286	1			43	1.44513	1		

Patient Health Questionnaire 9 (PHQ-9), Beck Depression Inventory II (BDI-II), Brief Symptom Inventory scale 4 (BSI-D), Generalized Anxiety Disorder Questionnaire (GAD-7), Brief Symptom Inventory scale 5 (BSI-A), Eating Disorder Inventory (EDI-II), Structured Interview for Anorexic and Bulimic Eating Disorders (SIAB), Body Mass Index (BMI).

Table S12. Spearman correlation of additional questionnaires for symptom severity of eating disorder pathology, anxiety, and depression before (T1) and after weight rehabilitation (T2) and their changes during inpatient treatment (longitudinal) with the relative abundance of taxa on family level.

	**T1**				**T2**				**Longitudinal**			
	**BDI-II**	**BSI-D**	**BSI-A**	**SIAB**	**BDI-II**	**BSI-D**	**BSI-A**	**SIAB**	**BDI-II**	**BSI-D**	**BSI-A**	**SIAB**
f__Bacteroidaceae	0.32			**0.44**								
f__Prevotellaceae									-0.34			
f__Ruminococcaceae					0.42	0.38						
f__Christensenellaceae												
f__Rikenellaceae											0.37	
f__Lachnospiraceae												
f__Bifidobacteriaceae									***0.45***	0.31		**0.45**
f__Oscillospiraceae											***0.42***	
f__Akkermansiaceae												
f__Tannerellaceae												
f__Sutterellaceae												
o__Rhodospirillales Order												
f__Erysipelatoclostridiaceae												
f__Veillonellaceae											0.3	
f__Acidaminococcaceae			-0.29	-0.32								
f__Coriobacteriaceae												
o__Gastranaerophilales Order			0.28	0.31							0.32	
f__Barnesiellaceae												
f__Desulfovibrionaceae							0.35					-0.3
f__[Eubacterium]_coprostanoligenes_group												
f__Marinifilaceae												
f__Muribaculaceae												
f__Acholeplasmataceae									0.38			0.38
o__Clostridia_UCG-014 Order				***-0.34***								
f__Enterobacteriaceae										-0.32	-0.34	

f__Pasteurellaceae												
f__Peptostreptococcaceae	-0.3											
f__Streptococcaceae												
o__Izemoplasmatales Order												
f__Monoglobaceae		0.29										
o__Clostridia_vadinBB60_group Order											**0.49**	
o__RF39 Order												
c__Clostridia Class												
f__Erysipelotrichaceae				***0.33***						-0.38	-0.31	
f__Clostridiaceae										-0.31		
f__Butyricicoccaceae					0.36							
f__Victivallaceae												
f__UCG-010												
f__Methanobacteriaceae						-0.34						
f__Eggerthellaceae												0.36
f__vadinBE97												
f__Anaerovoracaceae					0.36							
f__Lactobacillaceae				***0.35***		-0.32					-0.37	
f__Puniceicoccaceae			0.28									

The p-values were false discovery rate (FDR) adjusted. A FDR < 0.15 was considered as statistically significant. The table shows the spearman's rho in bold if FDR < 0.05, in bold italics if FDR > 0.05 but < 0.15 and in normal font if they did not withstand FDR adjustment. Beck Depression Inventory II (BDI-II), Brief Symptom Inventory scale 4 (BSI-D), Brief Symptom Inventory scale 5 (BSI-A), Structured Interview for Anorexic and Bulimic Eating Disorders (SIAB).

Table S13. Spearman correlation of additional questionnaires for symptom severity of eating disorder pathology, anxiety, and depression before (T1) and after weight rehabilitation (T2) and their changes during inpatient treatment (longitudinal) with Short Chain Fatty Acids (SCFA).

	T1				T2				Longitudinal			
	**BDI-II**	**BSI-D**	**BSI-A**	**SIAB**	**BDI-II**	**BSI-D**	**BSI-A**	**SIAB**	**BDI-II**	**BSI-D**	**BSI-A**	**SIAB**
Total SCFA												-0.37
Butyrate												
Acetate								-0.31				-0.42
Propionate								-0.32				-0.41
Valerate				-0.28								
Isovalerate				-0.32		0.34						
Isobutyrate												

A $p < 0.05$ was considered as statistically significant. The table shows the spearman's rho if $p < 0.05$. Beck Depression Inventory II (BDI-II), Brief Symptom Inventory scale 4 (BSI-D), Brief Symptom Inventory scale 5 (BSI-A), Structured Interview for Anorexic and Bulimic Eating Disorders (SIAB).
